# Emerging Roles of L-Type Voltage-Gated and Other Calcium Channels in T Lymphocytes

**DOI:** 10.3389/fimmu.2013.00243

**Published:** 2013-08-30

**Authors:** Abdallah Badou, Mithilesh K. Jha, Didi Matza, Richard A. Flavell

**Affiliations:** ^1^Equipe de recherche Environnement et Santé, Faculté Polydisciplinaire de Safi, Université Cadi Ayyad, Safi, Morocco; ^2^Trudeau Institute, Saranac Lake, NY, USA; ^3^Department of Cardiothoracic Surgery, Hadassah Medical Center, Jerusalem, Israel; ^4^Flavell Laboratory, Department of Immunobiology, Yale University School of Medicine, New Haven, CT, USA; ^5^Howard Hughes Medical Institute, New Haven, CT, USA

**Keywords:** Ca_v_1 channels, calcium channels, CD4 T cells, CD8 T cells, CRAC channel

## Abstract

In T lymphocytes, calcium ion controls a variety of biological processes including development, survival, proliferation, and effector functions. These distinct and specific roles are regulated by different calcium signals, which are generated by various plasma membrane calcium channels. The repertoire of calcium-conducting proteins in T lymphocytes includes store-operated CRAC channels, transient receptor potential channels, P2X channels, and L-type voltage-gated calcium (Ca_v_1) channels. In this paper, we will focus mainly on the role of the Ca_v_1 channels found expressed by T lymphocytes, where these channels appear to operate in a T cell receptor stimulation-dependent and voltage sensor independent manner. We will review their expression profile at various differentiation stages of CD4 and CD8 T lymphocytes. Then, we will present crucial genetic evidence in favor of a role of these Ca_v_1 channels and related regulatory proteins in both CD4 and CD8 T cell functions such as proliferation, survival, cytokine production, and cytolysis. Finally, we will provide evidence and speculate on how these voltage-gated channels might function in the T lymphocyte, a non-excitable cell.

## Introduction

T cells require Ca^2+^ for their development and function (1–4). A canonical pathway for Ca^2+^ entry into T cells has been described thus far. Accordingly, ligation of T Cell Receptor (TCR) leads to activation of phosphoinositide-specific phospholipase C (PLC)γ. PLCγ breaks down phosphatidylinositol-4,5-bisphosphate to generate inositol-1,4,5-trisphosphate (IP_3_) and diacylglycerol (DAG). IP_3_ activates the release of Ca^2+^ into the cytoplasm by binding to IP_3_ receptors (IP_3_R) located on the surface of internal Ca^2+^ stores, such as the endoplasmic reticulum (ER). Store-operated calcium (SOC) channels in the plasma membrane are then activated by the store depletion (5–7). A requirement for sustained signaling arises largely from the need to recruit and retain Nuclear Factor of Activated T cells (NFAT), a key transcriptional regulator of the IL-2 gene and other cytokine genes, in the nucleus (8).

There are several families of plasma membrane channels expressed in T cells. The most studied channels in lymphocytes are known as “calcium release-activated calcium” (CRAC) channels (5, 9, 10). A breakthrough in their characterization occurred after the identification of stromal interaction molecule (STIM), which is an ER-resident Ca^2+^ sensor, and ORAI/CRACM (CRAC modulator), which is their pore-forming subunit (11–14). The transient receptor potential (TRP) channels have also been detected in T cells and reported to be functionally involved in Ca^2+^ entry possibly after store depletion (15–17). Finally, evidence for the expression of P2X receptor channels on the plasma membrane and for their contribution to Ca^2+^ entry in lymphocytes was also shown (18–20).

## Expression of Ca_v_ Channels in T Cells

Ca_v_ channels are heteromultimers that are composed of a pore-forming α1 subunit, β regulatory subunit, and α2, γ, and δ subunits (21). The topology of the α1 pore subunit is predicted to have four repeated motifs (I–IV), each of which is hexahelical and contains a loop between the S5 and S6 transmembrane segments that forms the channel pore. The S4 transmembrane segments in each motif contain conserved positively charged amino acids that are voltage sensors and that move outwards upon membrane depolarization, thereby opening the channel (22).

Several studies, including our own, have shown that CD4^+^ and CD8^+^ T cells express high levels of the Ca_v_1 pore-forming subunit subfamily (Ca_v_1.1–1.4 or α1S, α1C, α1D, and α1F, respectively), but not Ca_v_2 (α1A, α1B, α1E) or Ca_v_3 (α1G, α1H, α1I) subfamilies (see Table 1); moreover they express these molecules at levels comparable with those in excitable cells (23–32).

**Table 1:** **Role of distinct Ca^2^^+^-permeable channels in T lymphocyte development and functions**.

Channel	Role in T lymphocytes			Evidence	Reference
	Development	Naive	Differentiated		
Ca_v_1.1	ND	Expression was detected in naïve CD4+ T cells and a role in TCR-mediated Ca2+ influx	Expression was detected in effector CD8+ T cells. Contribution in TCR-mediated Ca^2+^ entry and CTL effector functions	β4 and AHNAK1-deficient T cells express low levels of the Ca_v_1.1 protein	[(27) #3; (29) #15; (33) #14]
Ca_v_1.2 Ca_v_1.3	ND	No apparent expression	Involvement in TCR-mediated calcium influx in Th2 cells and in Th2 effector functions *in vitro* and *in vivo*	dihydropyridines antagonists and knockdown with Ca_v_1 antisense oligodeoxynucleotides	[(34) #6; (60) #195; (27) #3; (36) #280]
Ca_v_1.4	Involvement in thymic development	Requirement for TCR-induced calcium influx in naïve T cells	Requirement for CD4+ and CD8+ T cell immune responses	Ca_v_β3 KO mice and Ca_v_1.4 KO mice	[(31) #13; (32) #283]
		Essential for survival and naive T cell maintenance	
ORAI1	No apparent effect in ORAI1-deficient mice	No apparent effect in ORAI1-deficient mice	Involvement in TCR-mediated Ca^2+^ influx and effector functions (in T cells from SCID patients) and contribution to TCR-mediated Ca^2+^ influx and effector functions (in ORAI1-deficient T cells from mice)	T cell lines from SCID patients and primary murine T cells from ORAI1 KO mice	[(13) #211; (14) #212; (74) #249]
TRPC3	ND	ND	Contribution to TCR-dependent calcium influx suggested.	T cell lines and primary human T cells/overexpression and siRNA	[(16) #244; (82) #257]
TRPM2	No apparent effect in TRPM2-deficient mice	Reduced TCR-mediated proliferation	Contribution to production of pro-inflammatory cytokines after stimulation via TCR	TRPM2 KO mice	[(89) #266]
TRPM7	defect in T cell development in the thymus	ND	ND	TRPM7 KO mice	[(91) #268]
P2X7, P2X1, and P2X4 receptor channels	No apparent effect in P2X7 deficient mice and ND for P2X1 and P2X4	ND	Critical for TCR-dependent, ATP-mediated Ca^2+^ influx and downstream signaling events accompanying T cell activation	P2X7 receptor KO mice and siRNA for P2X7, P2X1, and P2X4 receptor channels. Jurkat cells and human peripheral CD4+ T cells were used	[(18) #270; (19) #276; (20) #271]

*In this table, we consider major Ca^2+^ permeable channels, which may contribute either directly or indirectly to TCR-mediated Ca^2+^ influx, development, initial activation of naïve T cells and effector functions in differentiated T cells. Our goal in distinguishing roles of different channels at different differentiation stages is to emphasize areas where more research efforts are needed in order to understand the contribution of these Ca^2+^ channels in T lymphocyte development and functions. Cav, voltage-gated Ca^2+^ channel; TRP, transient receptor potential; P2XR, P2X receptors; TCR, T cell receptor; Th, T helper; ND, not determined*.

We showed that the Ca_v_1.1 pore subunit is expressed in naïve CD4^+^ T cells and its expression is upregulated during primary stimulation of these cells (27, 29, 35). In CD8^+^ T cells, this subunit is only expressed in effector cells, late after primary stimulation and during secondary stimulation (31, 33).

The Ca_v_1.2 pore subunit is apparently not detected in naïve CD4^+^ or CD8^+^ T cells. In the CD4 compartment, effector Th2 CD4^+^ cells selectively express this subunit and it is not expressed in effector CD8^+^ T cells. In CD8^+^ T cells, its protein expression seems to be upregulated briefly during primary stimulation (usually peaks at day 3 or 4 after *in vitro* stimulation of CD8^+^ T cells using anti-CD3 and anti-CD28 coated plates) (33).

The Ca_v_1.3 pore subunit is expressed in effector Th2, but not in naïve, CD4^+^ T cells (36). Its mRNA was detected in naïve and effector CD8^+^ T cells but no information is yet available regarding the protein expression profile in these cells. Finally, the Ca_v_1.4 pore subunit is expressed in naïve CD4^+^ and CD8^+^ T cells (31, 32). Apparently T cells express all the rest of the Ca_v_ complex subunits, including the regulatory β subunits, γ subunits, as well as α2, and δ subunits. It is therefore likely that these cells express a fully functional Ca_v_ channel, possibly similar to the ones found in excitable cells (25, 27). Other studies have also shown that these channels are widely expressed in various other immune cell types, such as Dendritic cells (DC), B-lymphocytes, and monocytes (37–39).

In addition to the expression of a full Ca_v_ complex, other similarities exist between excitable and non-excitable cells in relation to the Ca_v_ pathway. In striated muscle, Ca_v_ channels, expressed on the plasma membrane, are physically linked to Ryanodine receptors (RyR), expressed in the Sarcoplasmic Reticulum (SR). During a process called excitation-contraction coupling (E-C coupling), depolarization of the t-tubule membrane (i.e., excitation) induces extracellular Ca^2+^ flow through Ca_v_ channels (which are gated by the function of their voltage sensor) that lead to activation of RyR channel in the SR membrane. The activation of RyR channels leads to massive Ca^2+^ release from the SR, which in turn initiates contraction (40). Therefore, unlike T cells, muscle cells first obtain Ca^2+^ from the extracellular space that initiates the entire process of Ca^2+^ release from intracellular stores.

It seems that T cells also express all the components necessary for such a mechanism described above, i.e., RyRs and Ca_v_ channels. Primary T cells express RyR2, and they upregulate its expression after treatment with stromal cell-derived factor 1 (SDF-1), macrophage-inflammatory protein-1 α (MIP1α), or TGF-β. Other hemopoietic cells also express RyRs (41, 42). RyRs, expressed in T cells, can be activated pharmacologically to mobilize Ca^2+^ from intracellular stores independently from IP_3_R (43). On the other hand, pharmacological blocking of RyRs in T cells results in reduced proliferation and IL-2 production (44). Knockdown of RyR3, the RyR that is expressed mainly by Jurkat T cells (primary T cells express RyR2 mostly), resulted in a significant reduction in Ca^2+^ entry in response to TCR cross-linking using anti-CD3 (45).

Finally, a recent study has suggested that, similar to excitable cells, store-operated Ca^2+^ entry via TCR stimulation precedes Ca^2+^ release from intracellular stores via IP_3_R and RyRs (46). Further studies are required to determine if Ca_v_ channels are associated with RyRs in T cells and what are their roles in T cell activation.

## Role of β Regulatory Subunits and Ca_v_1 Channels in T Cell Activation and Function

Numerous lines of evidence demonstrating the expression of Ca_v_ channels have indicated roles of these channels in T cell biology (see Table 1). A potential role for Ca_v_ channels in T cells became evident in mice with *lethargic* mutation, which arose spontaneously in the inbred mouse strain BALB/cGn in 1962. Homozygotes are recognizable at 2 weeks of age by ataxia, seizures, and lethargic behavior (47, 48). In 1997, Burgess et al. demonstrated that the ataxia and seizures in the lethargic mouse arise from a mutation of the β4 subunit gene (49). Neither full-length nor truncated β4 protein is expressed in the mutant mice (49). Interestingly, these mice experience an immunological disorder, including a defect in their cell-mediated immune response (50). β regulatory subunits, β1–β4, are crucial for normal Ca_v_ channel function (51), since they are required for the expression of functional channels at the plasma membrane (52), and modulate their biophysical properties by interacting with pore-forming α subunit (51). The mechanism of immune disorder described in these lethargic β4 mutant mice was unknown but of great interest since it implicitly supported the hypothesis that components of Ca_v_1 channels are expressed in immune cells and play a crucial role in the activation and function of immune cells.

We and others demonstrated that human and mouse T cells express regulatory β3 and β4 subunits (23–25, 27, 31, 33, 53). In 2006, we provided genetic evidence, for the first time, that CD4^+^ T cells deficient in either β3 and β4 are impaired in Ca^2+^ response, NFAT activation, and cytokine production (27). Interestingly, in the β4-deficient T cells, we have also detected a notable and specific suppression of the Ca_v_1.1 pore-forming α1 subunit protein. On the other hand, no significant effect was observed in the expression of the Ca_v_1.2 channel protein. This observation suggests that the deficiency observed in the β4-deficient mice might be due to the lack of expression of the Ca_v_1.1 channel (27). However, the exact mechanism of the requirement of multiple β regulatory subunits in effector T cell stage is still unknown.

In CD8^+^ T cells, we found that β3 is highly expressed in naïve and activated CD8^+^ T cells and β3 deficiency leads to enhanced apoptosis of naïve T cells and decrease in homeostatic survival of these cells (31). We found that the impaired Ca^2+^ influx in β3-deficient CD8^+^ T cells was associated with a lack of Ca_v_1.4 protein expression (31). The functional defect in both β4- and β3-deficient T cells reflected the contribution of these subunits to Ca_v_1 channel-dependent calcium response in T lymphocytes (27, 31).

Consistent with our findings (31), Omilusik et al. analyzed Ca_v_1.4-deficient mice and reported that CD4^+^ and CD8^+^ T cells from Ca_v_1.4-deficient mice had impaired homeostatic maintenance (32). β3 or Ca_v_1.4-deficient T cells also had increased rates of cell death (31, 32). Naive CD4^+^ and CD8^+^ T cells were shown to be dependent on Ca_v_1.4 function for SOCE, TCR-induced rises in cytosolic Ca^2+^ and downstream TCR signal transduction. The generation of antigen-specific T cell responses was altered in the absence of β3 or Ca_v_1.4 (31, 32) since these mice failed to mount an effective T cell response to antigen challenge, and this was associated with reduced effector function of CD8^+^ T cells (32).

Unexpectedly, we found that β3 and Ca_v_1.4 were associated with a T cell signaling complex in primary T cells that was not dependent on TCR stimulation, which suggested that a preformed complex of these proteins exists in naive T cells (31). Furthermore, we identified a fraction of Ca_v_1.4 as a lipid raft-resident Ca^2+^ channel protein (31). The reported interaction of Ca_v_1.4 with filamins in spleen cells (54) combined with our finding of its association with Lck and Vav highlight a Ca_v_ channel-dependent molecular architecture of a signaling complex in specialized microdomains of T cells. These observations further gain importance given the widely accepted model that the specificity, reliability, and accurate execution of signaling processes depend on tightly regulated spatiotemporal Ca^2+^ signals restricted to precise microdomains that contain Ca^2+^-permeable channels and their modulators (55, 56).

Similar to β3 deficiency, analyses of thymocytes lacking a functional Ca_v_1.4 channel revealed unperturbed or subtle changes in T cell compartment (31, 32). In thymus, the expression of various maturation and activation markers such as TCRβ, CD44, CD69, and CD62L were similar on Ca_v_1.4^-/-^ and WT double positive (DP) and TCRβ^+^ SP subpopulations (32). Ca_v_1.4-deficient SP thymocytes exhibited very moderate decreases in TCR- or thapsigargin-induced rises in cytosolic-free Ca^2+^ relative to WT. In contrast to thymocytes, Ca_v_1.4^-/-^ peripheral naive, and memory T cells were significantly impaired in TCR- or thapsigargin-induced rises in cytosolic-free Ca^2+^ compared to WT peripheral naive and memory T cells (32). This indicates the great complexity involved in Ca^2+^ regulation, dynamically changing with T cell differentiation, and suggests that differential responses are important for functional outcomes upon TCR engagement. These two independent studies indicated that Ca_v_1.4/β3 complex-mediated influx of Ca^2+^ from outside the cell probably induces a signaling cascade as well as contributes to tonic filling of intracellular Ca^2+^ stores critical for TCR survival signaling (31, 32).

## Differential Regulation of T Cell Survival by Ca_v_ vs. CRAC Channels

**Figure 1:**
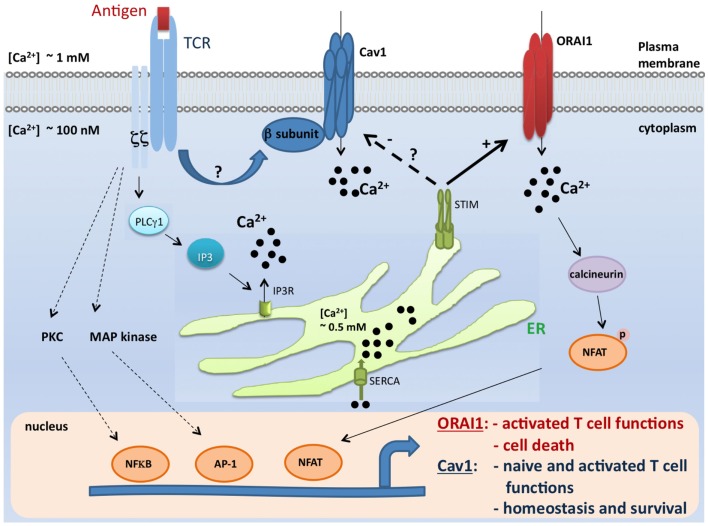
**A model for coordinated control of Ca_v_1 and ORAI1 channels in T lymphocytes**. Antigen encounter by T cells results in the activation of numerous pathways including the Ca^2+^ pathway. Mechanisms of Ca^2+^ influx through two major Ca^2+^-permeable channels, Ca_v_1 and ORAI1, are depicted in this scheme. During the course of biological functions that require activation of the STIM/ORAI pathway (such as effector functions and apoptosis), STIM1 blocks Ca_v_1 channel activity and all depending T cell functions. In contrast, this inhibitory effect would be lifted when Ca_v_1-dependent T cell functions (such as survival and naïve T cell activation) take place (27, 29, 31, 33, 32, 36, 57–59, 77, 78). It is, however, important to point out that the crosstalk described in this model was shown solely for Ca_v_1.2 channel, and no information is available to date for the relationship between STIM and other Ca_v_1 channels. TCR, T cell receptor; Ca_v_, voltage-gated Ca^2+^ channels; ER, endoplasmic reticulum; IP3, inositol-1,4,5-trisphosphate; SERCA, sarco-endoplasmic reticulum Ca^2+^-ATPase; STIM1, stromal interaction molecule 1; PLCγ1, phospholipase Cγ1; MAP kinase, Mitogen-activated protein kinase; PKC, protein kinase C; NFkB, nuclear factor kB; AP-1, activator protein-1; and NFAT, nuclear factor of activated T cells.

While β3^−/−^ or Ca_v_1.4^−/−^ naïve T cells die spontaneously (31, 32), it was surprising to find an enhanced T cell survival and proliferation in the absence of ORAI1/CRACM1 (57). CD4^+^ T cells from *Orai1*^-/-^ mice showed robust proliferation with repetitive stimulations and strong resistance to stimulation-induced cell death due to reduced mitochondrial Ca^2+^ uptake and altered gene expression of proapoptotic and antiapoptotic molecules. *Orai1^-/-^* mice showed strong resistance to T cell depletion induced by injection of anti-CD3 Ab. Furthermore, ORAI1-deficient T cells showed enhanced survival after adoptive transfer into immunocompromised hosts. Together, therefore these data suggest a unique requirement of Ca_v_1 calcium channels, not ORAI1/CRACM1 channel, in the survival, homeostasis, and proliferation of naïve T cells. While ORAI1/CRACM1 channels are undoubtedly required for the effector/late T cell functions (see Figure 1), others and our data also argue for a requirement for Ca_v_1 calcium channels in the effector stage of T cells (27, 31–33). Although, it is clear now that both types of calcium channels (Ca_v_1 and ORAI1/CRACM1) play critical roles in T cell biology, the present state of knowledge does not rule out a cross talk between Ca_v_1 and ORAI1/CRACM1 calcium channels at the effector stage of T cells where all different kinds of calcium channels (Ca_v_1.1, Ca_v_1.2, Ca_v_1.3, Ca_v_1.4, and ORAI1/CRACM1) are co-expressed and deficiencies in these channels show immune defects. Indeed, STIM1 was shown to reciprocally control Ca_v_1.2 and ORAI1 channels. While STIM1 activates the ORAI1 channel, it blocks Ca_v_1.2 channel activity (58, 59). When Ca_v_1.2 was introduced into Jurkat T cell lines expressing reduced levels of STIM1, the authors were able to measure a significant depolarization-induced increase in [Ca^2+^]i compared to WT Jurkat cells (59). This suggests that loss of STIM1 allowed Ca_v_1.2 activation in these cells. This was further confirmed by using shRNA to suppress STIM1. The regulation of Ca_v_1.2 by STIM1 occurs through direct interaction since by using co-immunoprecipitation, it was shown that these two proteins co-interact after overexpression but also at their physiological expression level in neuroblastoma cells. Furthermore, it was reported that STIM1 binds to the C terminal region of Ca_v_1.2 through its CRAC activation domain (CAD) (58, 59). These observations may explain how these two widely expressed channel families, Ca_v_1 and ORA1, could function in the same cell type to trigger different signaling pathways, potentially leading to the control of different functions (Figure 1).

## Role of Ca_v_ Channels in T Cell Differentiation and Inflammatory Disorders

Savignac et al. demonstrated that expression of Ca_v_1 channels was induced during Th2 cell differentiation (60). Agonists and antagonists for Ca_v_1 channels modulate the TCR-dependent increase in [Ca^2+^]_i_ and IL-4 production by Th2 cells, whereas they failed to alter the Th1 cell responses. The administration of nicardipine, a specific and clinically approved inhibitor for Ca_v_1 channels, was found beneficial in three models of Th2-mediated immunopathology but did not prevent experimental autoimmune encephalomyelitis (EAE), an experimental model of Th1-mediated autoimmune disease (60, 61). These studies highlighted that TCR-dependent calcium signaling differs between Th2 and Th1 cells and suggested an important role of Ca_v_1 channels in the selective regulation of [Ca^2+^]_i_ on stimulation through the TCR in Th2 cells. It is important to note that drugs targeting Ca_v_1 channels may be beneficial in the treatment of pathologies associated with Th2 cell-mediated immunopathology.

Further, it is reported that differentiation in Th2 cells but not in Th1 cells was associated with the up-regulation of Ca_v_1.2 and Ca_v_1.3 channels both at the mRNA and protein level (36). Depletion of Ca_v_1.2 and Ca_v_1.3 expression by antisense oligodeoxynucleotides in T cells reduced TCR-induced Ca^2+^ influx in Th2 cells, attenuated IL-4 production and reduced airway inflammation in a mouse model of allergic asthma (36). Moreover, ovalbumin (OVA)-specific transgenic Th2 cells transfected with Ca_v_1-specific antisense (Ca_v_1AS) oligodeoxynucleotides were no longer able to induce asthma on adoptive transfer in BALB/c mice given intranasal OVA. The intranasal administration of Ca_v_1AS at the time of intranasal challenge with OVA was effective in active experimental asthma, preventing airway inflammation, Th2 cell activation in the lung draining lymph nodes, and airway hyperreactivity (36).

## Mechanism of Ca_v_1 Channel Mediated Regulation of Ca^2+^ Signaling in T Lymphocytes

Others and we demonstrated the presence and significance of Ca_v_1 channels in T cells (see Table 1) (23, 25, 27, 31, 33, 53). However, it is not known how these Ca_v_1 channels open in T cells to conduct calcium. In excitable cell types, Ca_v_ channels conduct Ca^2+^ upon depolarization (62, 21). The basic question here is whether Ca_v_1 channels are activated by TCR stimulation or by depolarization. From a physiological standpoint, T cells should respond only to antigen stimulation through cognate TCR. A voltage-dependent opening in the absence of TCR dependence would lead to a random opening of Ca_v_ channels and subsequent activation of T cells, which could lead to immune activation in the absence of antigen. Unlike excitable cells, T cells migrate and roam the body through variable extracellular environments and tissues with various ion concentrations. It is therefore conceivable that Ca_v_ channels expressed by T cells have developed a more specific control of their opening than mere voltage sensing. Notably, Ca_v_1.4, as well as Ca_v_1.3, has been found to have low activation thresholds that do not require strong depolarization for their activation (63). Earlier surprising findings showed that Ca_v_1.3 channels can be activated at voltages of approximately -60 mV under physiological calcium concentrations (64).

Since Ca_v_1 channels are expressed in T lymphocytes before and after TCR stimulation (23, 25, 27, 31, 33, 53), we tested the susceptibility of T cell Ca_v_ channels to depolarization induced by KCl. Artificial depolarization of CD4^+^ T cells, which have been differentiated under Th1 (IL-12 plus anti-IL-4), Th2 (IL-4 plus anti-IFNγ), or Th0 (no cytokine) conditions, with KCl did not lead to calcium influx (27). KCl was used at 40 mM, a dose that induces a significant depolarization of T cells (27). However, under the same conditions and as expected, KCl triggered a transient calcium response in the C2C12 skeletal muscle excitable cell line as previously reported (65). In addition, all four groups of cells, Th0, Th1, Th2, and C2C12, were able to mount a calcium response after stimulation with the calcium ionophore, ionomycin (27). In agreement with our findings, other studies also have shown that treatment of T cells with KCl does not lead to calcium entry (25, 66) and in fact KCl addition seems to inhibit proliferation and IL-2 production (67). These observations demonstrate that, unlike in excitable cells, depolarization of T cells does not induce Ca_v_ channel opening.

## Other Ca^2+^-Permeable Channels Expressed by T Lymphocytes

The encounter of peptide-antigen presenting cell (APC) by naïve T cells induces a quick increase of intracellular calcium concentration in T lymphocytes (4). This calcium increase could be sustained for hours at levels higher than basal standards in order to mediate appropriate T lymphocyte functions such as activation, proliferation, expression of various activation-associated genes such as cytokines and chemokines (4, 7, 68). During their maturation stages, naïve T lymphocyte differentiate into distinct T cell subpopulations (such as Th1, Th2, Th17, and Treg), all of which require calcium signal. In light of these multitude and specific functions governed by T cells, it is logical to discover the expression, by these cell types, of various plasma membrane calcium channels, or even different levels of expression of the same channel at different stages of differentiation. In this section, we review the role of three major families of Ca^2+^ permeable channels expressed by T lymphocytes, SOC channels, TRP channels, and P2X receptor channels.

### Store-operated calcium channels

One well studied mechanism of calcium entry into T cells is the store-operated Ca^2+^ (SOC) entry process. This mechanism was suggested by Putney (69). In this study, the authors presented evidence showing that the Ca^2+^ released from ER stores could “directly” induce Ca^2+^ influx through plasma membrane calcium channels in cells that are non-excitable (69). Numerous and independent electrophysiological studies showed that T cells indeed express channels that can be opened in response to store depletion by distinct stimuli (4, 5, 70). These channels have been designated CRAC channels in T cells, and have been extensively characterized at the electrophysiological level (4, 71) and are distinguished by a high selectivity for Ca^2+^ and a low conductance (4, 71). However, the molecular identity of the channels and their related regulatory proteins had remained unknown. In the year 2005, it has first been proposed, using RNA interference (RNAi)-based screen, that STIM 1, a conserved protein, is required for SOC influx both in *Drosophila* S2 cells and in Jurkat T cells (72). In a second study, by generating a point mutation in the STIM1 Ca^2+^ binding domain, it has been proposed that STIM1 operates as a Ca^2+^ store sensor, which functions by connecting Ca^2+^ store depletion to Ca^2+^ influx (11). In 2006, by using genome-wide approaches designed to identify regulators of store-operated Ca^2+^ entry, three separate groups have proposed a protein containing four transmembrane segments, ORAI1 (also named CRACM1) as the putative CRAC channel, or an essential component or related regulatory protein of the CRAC channel (13, 73, 74). It was still not clear, at this stage, whether ORAI1 forms the CRAC channel itself. Using site-directed mutagenesis, three additional studies have shown that it is indeed ORAI1 itself that forms the Ca^2+^ selectivity filter of the CRAC channel complex, providing strong evidence that ORAI1 is the pore-forming subunit of the CRAC channel (14, 75, 76). In addition, the protein ORAI1 was proposed as the prototypical CRAC channel, especially after the discovery that human patients presenting with a SCID disease lack functional CRAC channels and SOCE in T cells (13). However, ORAI1 deficiency in mice resulted surprisingly in no defect in T cell development in the thymus, no defect in T cell proliferation and only a partial inhibition of IL-2 and IFNγ production (77). In contrast, these mice exhibited a major defect in mast cell effector functions (77). It was also reported, in a second independent study, that T cell development is normal in ORAI1^−^/^−^ mice and that ORAI1-deficient naive CD4+ T cells and CD8+ T cells show no significant decrease of SOC influx after stimulation by thapsigargin or by anti-CD3 mAb (78). Consistently, ORAI1-deficient naive T cells exhibited normal proliferation upon stimulation with anti-CD3 and anti-CD28 mAbs (78). However, ORAI1 seemed to be of more importance to differentiated CD4+ and CD8+ T cells. Indeed, the impairment in Ca^2+^ influx in the absence of ORAI1 was most apparent in Th1 cells, followed by CTLs and then Th2 cells (78). Furthermore, when cytokine production was assessed, only a partial inhibition was observed in differentiated CD4+ and CD8+ T cells from ORAI1^−/−^ mice (78). Interestingly, when cyclosporine A was applied, cytokine production was completely abolished (78), indicating that other calcium/calcineurin-dependent, ORAI1-independent pathways are involved in this cytokine production in differentiated CD4 and CD8 T cells. The lack of a major contribution of ORAI1 is likely not due to a compensatory process by ORAI2 or ORAI3. In fact, while reconstitution with ORAI1 restored SOC influx in differentiated ORAI1-deficient T cells, reconstitution with ORAI2 protein showed no effect, and reconstitution with ORAI3 exhibited only a small SOC influx upon stimulation with thapsigargin but not anti-CD3 mAb (78). These observations suggest that ORAI1 is dispensable for T cell development and for initial intracellular calcium increases detected in naïve T cells upon the initial antigen encounter (see Table 1; Figure 1). However, ORAI1 is likely to contribute at least partially to CD4 and CD8 effector functions (see Table 1). This discovery is perhaps not completely surprising as the discovery phase of research on CRAC channels derived from studies in cell lines such as Jurkat, which are more similar, to some extent, to differentiated effector T cells rather than naive primary T cells. This observation also suggests that Ca^2+^ channels other than CRAC proteins are likely involved in T cell functions.

### Transient receptor potential channels

Before the discovery of ORAI1 as the main channel responsible for SOC influx in T cells, members of the TRP family were considered as key candidates for T cell calcium channels. In human cells, TRP superfamily of channels can be classified into 7 subfamilies (TRPC, TRPV, TRPM, TRPA, TRPN, TRPP, and TRPML) with a total of 27 cation channels (79). These channels, which share six transmembrane domains, form ion-conducting proteins that are mostly non-selective and permeable to several cations, including Ca^2+^ and Na^+^ (80). TRP channels can be activated via diverse mechanisms. In fact, some TRP channels could respond to stimuli ranging from heat to natural product compounds, pro-inflammatory agents, and exocytosis (79). TRPC, TRPM, and TRPV seem to be the major subfamilies expressed by murine (81) and human T cells (82) (see Table 1). In 2003, Hoth and colleagues, by analyzing mutant T cell lines exhibiting defects in Ca^2+^ entry and Ca^2+^-dependent gene expression (83), suggested an alteration of TRPC3 gene in these mutant cell lines relative to wild type cells. When the wild type TRPC3 gene was reintroduced in mutant cell lines through transient transfection, it was able to restore TCR-mediated Ca^2+^ influx. It was then concluded that TRPC3 channel contributes to TCR-induced Ca^2+^ entry into T cells, and is therefore critical for Ca^2+^-dependent activation of T cells (16). In this study, the authors used cell lines and overexpression approaches, and therefore, the conclusions needed to be confirmed in a more physiological system. A few years later, using murine immune cells the expression profile of diverse subsets of TRPC, TRPV, and TRPM was reported (81). Similarly, consistent mRNA expression of TRPC1, TRPC3, TRPV1, TRPM2, and TRPM7 was detected in primary human CD4+ T cells purified from healthy donors. TRPC3 and TRPM2 transcripts were upregulated after stimulation via TCR; and knockdown of TRPC3 channel by siRNA showed that this channel may contribute to Ca^2+^-dependent proliferation of primary T cells (82). Another study pointed out a significant role of TRPC5 channel in the mechanism of effector T cell suppression by Treg cells (84). Interaction of these two cell types was described as involving cross-linking of GM1 ganglioside (expressed by effector T cells) by galectin-1 (expressed by Treg cells); and the TRPC5 channel was shown to be involved in this regulatory process. In this paper, the authors described the up-regulation of TRPC5 channel transcript, but not TRPC4, in effector murine CD4, and CD8 T cells relative to naïve T cells (84). They also showed that knockdown of TRPC5 channel in effector T cells by short hairpin RNA inhibited both contact-dependent inhibition of effector T cell proliferation and galectin-1-induced Ca^2+^ influx (84). TRPM2 forms non-selective Ca^2+^-permeable cation channel. This channel is expressed in the brain but also in immune cells (85–87) and it can be opened by the intracellular messenger, adenosine 5′-diphosphoribose (ADPR) (85–87). One of the first reports on the role of TRPM2 (formerly LTRPC2) channel in calcium influx in immune cells demonstrated that the TRPM2 channel mediates Ca^2+^ influx into monocytes (86). This report showed that ADPR and nicotinamide adenine dinucleotide (NAD) can directly stimulate TRPM2 channel activity to mediate Ca^2+^ entry (86). A key question is whether these second messengers, NAD and/or ADPR, are involved in this process upon receptor stimulation. Guse and colleagues showed that indeed intracellular ADPR concentrations are increased upon stimulation of Jurkat T cells by ConA; and that this messenger mediates Ca^2+^ influx through TRPM2 channels (87). In this study, the authors also showed that inhibition of ADPR formation or knockdown of TRPM2 both inhibited this stimulation-dependent TRPM2-mediated Ca^2+^ influx (87). By modifying intracellular NAD concentration and using siRNA knockdown, another recent study similarly emphasized the role of NAD and ADPR in mitogen-induced Ca^2+^ rise in human T lymphocytes through the involvement of TRPM2 channels (88). Subsequently, using TRPM2-deficient mice, it was shown that this channel contributes to T lymphocyte proliferation and production of pro-inflammatory cytokines after stimulation via TCR (89). When evaluated *in vivo*, TRPM2^−/−^ mice displayed amelioration in EAE development. The authors attributed this improved EAE phenotype to reduced T cell effector functions and proposed TRPM2 channel as a potential therapeutic target (89).

Initial evidence for a role of the TRPM7 channel in immune cells emanated from its disruption in DT-40 B cell lines (90). TRPM7 deficient cells exhibited a defect in proliferation and required elevated extracellular Mg^2+^ for their survival (90). In a related interesting study, Clapham and colleagues used lck-Cre mice, since TRPM7^−/−^ mice died prenatally, to selectively delete TRPM7 in T cells. Surprisingly, TRPM7flox^−/−^ Lck-Cre mice displayed a notable defect in T cell development in the thymus. The authors detected a block in transition from the double negative (CD4 − CD8−) to DP (CD4 + CD8+) stage in TRPM7 deficient thymocytes. As a result, both the number and the percentage of T cells in the periphery are reduced. Interestingly, TRPM7 deficient thymocytes did not show any significant defect in Mg^2+^ uptake. And using inductively coupled plasma mass spectrometry, the authors showed that total Mg^2+^ concentration in wild type and deficient T cells is similar suggesting that TRPM7 is dispensable for cellular Mg^2+^ homeostasis in T cells (91). TRPM7 is a channel protein permeable to Ca^2+^ and Mg^2+^ but also contains a regulatory serine-threonine kinase domain in the same structure (92). A role for the kinase domain is likely to be also excluded. In fact, a recent study showed that the defect of TRPM7 deficient T cells in Fas-mediated apoptosis depends on its activity as a channel rather than a kinase (92). Therefore, after the involvement of Mg^2+^ and the kinase domain in this process have been excluded, the question arises as to whether the effects of the TRPM7 channel on T cell development are related to Ca^2+^.

In addition to TRPC and TRPM channels, other TRP channels such as TRPV1 and TRPV2 appear to show an interesting and consistent expression profile in primary human T cells (82), however, their role in T cell function is still elusive.

### P2X receptor channels

P2 receptors are broadly distributed in many cell types. Two distinct subfamilies have been described, the G-protein-coupled seven-transmembrane P2Y receptors and the ligand-gated P2X receptors (P2XR) (18). There are seven mammalian P2X receptor members (P2X1–7). These proteins form non-selective cation channels that are gated by extracellular ATP to allow influx of cations including Ca^2+^, and Na^2+^. In T lymphocytes, three distinct P2X members have been suggested to contribute to calcium entry in human T cells, P2X1, P2X4, and P2X7 (20) (see Table 1). One of the first reports to suggest a potential expression of ATP-gated receptor channels on T cells was published in 1996. In this article, it was shown that extracellular ATP (ATPe) was able to induce intracellular Ca^2+^ concentration increases in PBLs and purified human T cells (93). ATPe exhibited also a synergistic effect with PHA and anti-CD3 mAb on PBL proliferation. It was suggested, in this study, that the ATP-mediated Ca^2+^ influx and the ATP contribution in proliferation were both dependent on P2X and/or P2Z receptors since these effects were blocked using oxidized ATP (oATP), a covalent blocker of these two channels (93). In other reports, it was shown that ATPe was able to induce thymocyte apoptosis (94); and that the biochemical and morphological changes induced by ATPe and leading to apoptosis, are preceded by a rapid intracellular calcium increase (94). It was then documented that P2X7 receptor is critical for apoptosis of BALB/c thymocytes induced by ATPe (18). In fact, the potent P2X7 receptor agonist, benzoylbenzoyl-ATP, was able to mimic the ATPe effect. Furthermore, two P2X7 receptor antagonists (oATP and pyridoxalphosphate-6-azophenyl-2′,4′-disulfonic acid) inhibited the effect of ATPe. However, notable evidence emanated from the use of thymocytes prepared from P2X7R^−/−^ mice, where ATPe-induced apoptosis was completely abolished (18). Interestingly, ATPe could also induce activation of T cells (19, 95); and it appears that whether cells will undergo apoptosis or activation would depend on the level of expression of the P2X7 receptor and on concentrations of ATPe (19, 95). High concentrations of ATPe induce apoptosis, in contrast, lower ATPe doses closer to those secreted in an autocrine or paracrine manner would induce T cell activation (19). Indeed, Junger and colleagues showed that P2X7 receptors are critical for TCR-mediated Ca^2+^ influx and downstream signaling events accompanying T cell activation. The authors were able to reveal secretion of ATP (<100 μM) by Jurkat cells after TCR stimulation. Subsequently, they showed that released ATPe activates P2X7 receptors, in an autocrine manner, contributing to Ca^2+^ influx, which induces T cell activation via the activation of NFAT and IL-2 gene transcription (19). In addition to P2X7 receptor, the expression and involvement of two other critical members, P2X1 and P2X4, in calcium entry and T cell activation was reported (20). In this study, it was shown that P2X1, P2X4 receptors and pannexin-1 hemichannels translocate to the immunological synapse of activated T cells. Inhibition of pannexin-1, using the gap junction inhibitor carbenoxolone, resulted in the inhibition of TCR-mediated ATP release, Ca^2+^ influx and T cell activation. Similarly, inhibition or silencing of P2X1 and P2X4 receptors suppresses Ca^2+^ entry and subsequent signaling events leading to T cell activation, such as NFAT activation and IL-2 gene induction (20). These reports indicate that P2X1, P2X4, and P2X7 receptors play critical roles in TCR-mediated Ca^2+^ signal amplification upon stimulation of T lymphocytes.

## Perspectives and Concluding Remarks

Calcium ion is a critical and universal second messenger, which is involved in T lymphocyte function at various stages including development, survival, activation, differentiation, cytokine production, and cell death. In this review, we presented our views on the crucial role played by L-type Ca_v_1 channels in T cells. We also summarized the important discovery of the main elements controlling Ca^2+^ entry through CRAC channels in T cells, STIM, and ORAI. The contribution of other Ca^2+^ entry pathways such as the TRP family of channels and ligand-gated P2X receptors was also taken into consideration. Collectively, data reviewed in this manuscript show that T lymphocytes express a considerable number of Ca^2+^ permeable channels (see Table 1), which highly likely communicate together in order to regulate development and distinct functions of T cells. However, many questions still remain to be answered. While there is no doubt for a role of Ca_v_1 channel proteins in contributing to Ca^2+^ entry in T cells, it has still not been established that it is the Ca_v_1 pore-forming protein that conducts Ca^2+^ after TCR stimulation. Site-directed mutagenesis experiments could answer this important question. We also have presented evidence showing that Ca_v_1 channels expressed by T cells are not voltage-sensitive and contribute to Ca^2+^ entry after TCR stimulation (27, 29, 31, 32). How Ca_v_1 channels are gated after TCR stimulation is still not clear. Another major point is how different Ca_v_1, CRAC, TRP, and P2XR subsets contribute, physiologically, to development of T cells, but especially to their differentiation into various effector T cell subpopulations. As shown with distinct subsets of Ca_v_1 family of channels, the repertoire of Ca^2+^ channels operating in T cells changes during various stages of differentiation. A more profound study of the expression level of various channels after TCR stimulation, at various differentiation stages and under physiological conditions, will be of major interest. We believe that it will be of importance, therapeutically, to target a channel that is expressed at a specific stage on a specific T cell subpopulation rather than robust blockage of the entire immune system, which leads to major side effects. We also need to uncover factors that are implicated in physiological regulation of these channels. Ultimately, it will be crucial to understand how all these channels interact with each other to finely regulate T lymphocyte functions.

## Conflict of Interest Statement

The authors declare that the research was conducted in the absence of any commercial or financial relationships that could be construed as a potential conflict of interest.
